# Update on the Pancreas: Disease Mechanisms and Therapeutic Strategies

**DOI:** 10.1002/ueg2.12733

**Published:** 2024-12-12

**Authors:** Iris J. M. Levink, Alberto Balduzzi, Lumir Kunovsky, Albrecht Neesse

**Affiliations:** ^1^ Department of Gastroenterology and Hepatology Erasmus MC University Medical Centre Rotterdam Netherlands; ^2^ Department of Surgery, Dentistry, Paediatrics and Gynaecology Unit of General and Pancreatic Surgery The Pancreas Institute Verona University of Verona Verona Italy; ^3^ 2nd Department of Internal Medicine ‐ Gastroenterology and Geriatrics University Hospital Olomouc and Faculty of Medicine and Dentistry Palacky University Olomouc Olomouc Czech Republic; ^4^ Department of Surgery University Hospital Brno and Faculty of Medicine Masaryk University Brno Czech Republic; ^5^ Department of Gastroenterology and Digestive Endoscopy Masaryk Memorial Cancer Institute Brno Czech Republic; ^6^ Department of Gastroenterology Gastrointestinal Oncology and Endocrinology University Medical Centre Göttingen Göttingen Germany

The pancreas (Greek: κρέας; meat) is an important yet enigmatic organ located at the intersection of critical abdominal vessels and neighbouring organs. The absence of a defined capsule facilitates spread of infections or malignant cells from the diseased pancreas into the abdominal cavity and into lymphatic and blood vessels, presenting significant challenges for medical intervention. Its structural and anatomical complexity, coupled with its dual exocrine and endocrine functions, yields the pancreas as a focal point of intense medical research and clinical complexity.

Recently, remarkable advances in both clinical and molecular research have dramatically expanded our understanding of pancreatic diseases. Notwithstanding these substantial insights, a translational gap remains between scientific discovery and improved patient outcomes, underscoring the need for continued, sophisticated research that bridges fundamental scientific understanding with transformative clinical strategies.

In this special issue, a panel of international experts was assembled to provide readers with a comprehensive overview and a future perspective on current pancreatology.

Early detection of pancreatic cancer and adequate risk stratification of premalignant cystic neoplasms are of major clinical relevance. Artificial intelligence (AI), particularly machine learning (ML) and deep learning (DL), may enhance diagnostic precision, and support not only in pancreatic lesion detection but also differentiation. Podina et al. explain the basic principles of AI, guiding us away from the time‐consuming manual imaging segmentation to rapid AI‐based segmentation of CT, magnetic resonance (MR) and even endoscopic ultrasound (EUS) highlighting opportunities and challenges of AI and its integration into the healthcare system [1]. Meziani et al. performed a systematic review and meta‐analysis showing that stable‐sized, low‐risk intrapapillary mucinous neoplasms (IPMNs) are associated with low progression rate; for these ‘trivial cysts’ surveillance can potentially be de‐intensified and even discontinued, a strategy that is increasingly implemented in national and international guidelines [2].

Although there is currently no causal anti‐inflammatory treatment available for acute pancreatitis (AP), diagnostic assessment and treatment of the underlying aetiology and subsequent complications are crucial for patient outcomes. Beij et al. have come up with a true physician's manual that summarizes recommendations on the clinical management of AP. *Hands‐down* and *less‐is‐more* are key messages based on practice‐changing randomized controlled trials [3]. Additionally, the article by Borbély et al. provides a state‐of‐the‐art summary of the diagnosis and treatment of splanchnic vein thrombosis (SVT) in patients with pancreatitis and other pancreatic diseases [4]. Akshintala et al. have compiled an exciting overview of mechanisms that cause post‐ERCP pancreatitis (PEP) and describe conventional, as well as novel experimental tools, to prevent its development [5].

Chronic pancreatitis (CP) is a frequent cause of hospitalization and requires endoscopic, surgical, and nutritional expertise. Cardinal von Widdern et al. provide a practical overview of preventive and behavioural early interventions with a particular focus on pancreatic exocrine insufficiency, diabetes mellitus, pain, and pancreatic fluid collections [6]. Dankha et al. show that treatment of painful chronic pancreatitis is s truly multidisciplinary endeavour reminding us that early surgery should be considered in painful CP [7].

Autoimmune pancreatitis has a distinct pathophysiology and therefore requires a whole different treatment approach with glucocorticoids and other immunosuppressive drugs. Lanzillotta et al. provide a clear treatment algorithm for the diagnosis and treatment of both type 1 and 2 autoimmune pancreatitis whilst also offering insights into research perspectives and unmet needs of the rare disease [8].

Finally, malignant tumours of the pancreas remain a major challenge due to increasing incidence rates, particularly in young patients, poor outcomes, and overall survival rates. Thus far, for pancreatic ductal adenocarcinoma (PDAC), treatment personalization has been challenging due to difficulties in tumour acquisition, limited availability of accurate biomarkers, a complex tumour microenvironment, and hampered response to targeted‐ and immunotherapy. Organoids have emerged as a potentially powerful tool for treatment personalization recapitulating morphological and genetic patient‐related features of the tumour. Beutel et al. summarize the application of patient‐derived organoids in (pre) clinical practice, and how these can overcome the challenges of precision medicine within the field of pancreatic cancer [9].

Neuroendocrine neoplasms of the pancreas (pNETs) are rare and have a better prognosis. Tacelli et al. show that EUS provides the possibility to perform elastography, contrast‐enhanced EUS, and tissue acquisition in pNETs, and may provide an interventional treatment modality, as an alternative to systemic treatments, in a highly selected subgroup of patients [10].

We hope that you enjoy reading this special issue on pancreatic diseases as much as we did creating it. While challenges remain, the innovations being developed hold significant promise for a continued translation into our clinical routine and finally improved patient care.

## Conflicts of Interest

The authors declare no conflicts of interest.

## Data Availability

The data that support the findings of this study are available from the corresponding author upon reasonable request.
